# Systematic Review of the Diagnostic Validity of Brief Cognitive Screenings for Early Dementia Detection in Spanish-Speaking Adults in Latin America

**DOI:** 10.3389/fnagi.2020.00270

**Published:** 2020-09-04

**Authors:** Nilton Custodio, Lissette Duque, Rosa Montesinos, Carlos Alva-Diaz, Martin Mellado, Andrea Slachevsky

**Affiliations:** ^1^Research Unit, Instituto Peruano de Neurociencias, Lima, Peru; ^2^Cognitive Decline and Dementia Diagnostic and Prevention Services Unit, Instituto Peruano de Neurociencias, Lima, Peru; ^3^Neurology Department, Instituto Peruano de Neurociencias, Lima, Peru; ^4^Neuromedicenter Adult Day Care Center, Quito, Ecuador; ^5^Facultad de Ciencias de la Salud, Universidad Científica del Sur, Lima, Peru; ^6^Neuropsychology and Clinical Neuroscience Laboratory (LANNEC), Physiopathology Department, ICBM, Neurosciences and East Neuroscience Departments, University of Chile School of Medicine, Santiago, Chile; ^7^Geroscience Center for Brain Health and Metabolism (GERO), University of Chile School of Medicine, Santiago, Chile; ^8^Memory and Neuropsychiatric Clinic (CMYN), Neurology Department, Del Salvador Hospital and University of Chile School of Medicine, Santiago, Chile; ^9^Neurology Unit, Department of Medicine, Alemana Clinic, Universidad del Desarrollo, Santiago, Chile

**Keywords:** cognitive screening, dementia, Alzheimer's disease, mild cognitive impairment, MoCA, Latin America

## Abstract

**Objectives:** The aim of this study was to evaluate the validity of brief cognitive screening (BCS) tools designed to diagnose mild cognitive impairment (MCI) or dementia in Spanish-speaking individuals over the age of 50 years from Latin America (LA).

**Methods:** A systematic search of titles and abstracts in Medline, Biomed Central, Embase, Scopus, Scirus, PsycINFO, LILACS, and SciELO was conducted. Inclusion criteria were papers written in English or Spanish involving samples from Spanish-speaking Latin American individuals published until 2018. Standard procedures were applied for reviewing the literature. The data related to the study sample, methodology, and procedures applied, as well as the performance obtained with the corresponding BCS, were collected and systematized.

**Results:** Thirteen of 211 articles met the inclusion criteria. The studies primarily involved memory clinic-based samples, with the exception of two studies from an adult day-care center, one from a primary care clinic, and one from a community-based sample. All the studies originated from five of the 20 countries of LA and all used standardized diagnostic criteria for the diagnosis of dementia and MCI; however, the diagnostic protocols applied differed. Most studies reported samples with an average of 10 years of education and only one reported a sample with an average of <5 years of education. No publication to date has included an illiterate population. Although the Montreal cognitive assessment (MoCA) is the most widely-used BCS tool in LA, it is significantly influenced by education level.

**Conclusions:** Although evidence is still limited, the findings from studies on LA populations suggest that MoCA requires cultural adaptations and different cutoff points according to education level. Moreover, the diagnostic validity of the INECO frontal screening (IFS) test should be evaluated in populations with a low level of education. Given the heterogeneity that exists in the levels of education in LA, more studies involving illiterate and indigenous populations are required.

## Introduction

Dementia has become a public health priority in Latin America (LA) owing to the increasing life expectancy of the population, which has led to escalating rates of neurological disorders (Custodio et al., 2017c). The number of people with dementia in LA is expected to rise fourfold by 2050 (Parra et al., 2018). By 2020, it is estimated that 89.28 million people will be living with dementia in low- and middle-income countries (LMICs), compared with 42.18 million in high-income nations (Bongaarts, 2009). Timely diagnosis is one of the most promising strategies for addressing dementia and reducing patient and caregiver morbidity (Watson et al., 2018). However, dementia is significantly underdiagnosed, especially in LMICs. Indeed, some studies have suggested that only 3% of dementia patients are diagnosed by their primary care providers in these countries (Chong et al., 2016), and it is estimated that 77% of dementia cases in Brazil go undiagnosed (Nakamura et al., 2015). Barriers to dementia diagnosis that are particularly relevant to LA include inadequate physician training (Olavarría et al., 2016; Mansfield et al., 2019), especially among primary care providers (Saxena et al., 2007; Parra et al., 2018); lack of knowledge about different types of dementia, such as frontotemporal dementia (FTD) (Gleichgerrcht et al., 2011; Custodio et al., 2018a); language barriers; the stigma associated with age-related health problems; insufficient access to healthcare; a lack of diagnostic protocols; and scarcity of neuropsychological services (Custodio et al., 2017c; Parra et al., 2018).

The brief cognitive screen (BCS) is an instrument used to detect signs of dementia that does not include caregiver or information interviews. BCSs can be a useful tool for timely dementia detection, and can be used both for screening the general geriatric population and confirming the presence of a cognitive disorder in people with clinical suspicion of dementia (Brown, 2014; Velayudhan et al., 2014).

BCSs are routinely used in clinical practice, both to detect cognitive decline or dementia and to monitor disease evolution and treatment response (Carnero-Pardo, 2014). A positive screening result can provide a “wake-up call” to providers and caregivers that a detailed cognitive evaluation is indicated (Zucchella et al., 2018). BCSs are crucial for identifying the presence of a cognitive syndrome and initiating the diagnostic process, which may include supporting tests such as blood tests, brain imaging, and eventually a formal neuropsychological evaluation (Brown, 2014; Custodio et al., 2018b; Parra et al., 2018).

An appropriate BCS should fulfill the following characteristics: Administration time should be brief (no longer than 5 min for primary care or 10 min for specialist care) and require minimal additional material; the tools should be user-friendly and easy to administer and score; studies that provide norms and show that the instrument has appropriate psychometric properties should be available (Carnero-Pardo, 2014); the tool should be applicable to all patients regardless of education level, sociodemographic characteristics, and ethnic or cultural group; finally, it is important that the BCS can be administered by any professional (specialists, primary care physicians, or other nonmedical personnel) and in any location (home, outpatient office, or hospital). Evidence suggests that physicians and primary care nurses, if adequately trained, are capable of performing dementia screening with reasonable accuracy, using clinical observations and routine tests, during a typical office visit (Prince et al., 2011). With a few hours of training, community health workers in LMICs can identify signs of dementia with a positive predictive value of 66% (Ramos-Cerqueira et al., 2005).

The mini-mental state examination (MMSE) is the most widely used of the available BCS tools, and has been validated in LA (Ostrosky-Solís et al., 2000; Rosselli et al., 2000; De Beaman et al., 2004; Franco-Marina et al., 2010). However, this method has several drawbacks. Administration is not standardized; the cultural and socioeconomic characteristics of the patient may bias scores, and the tool can detect dementia only in individuals with at least 5 years of education; the tool does not measure executive function; finally, the MMSE can only detect moderate or advanced dementia, and is not sensitive to early-stage Alzheimer's disease dementia (ADD) or non-Alzheimer's dementias (Dubois et al., 2000). Recently, other BCSs have been proposed as substitutes, such as the Montreal cognitive assessment (MoCA) (Gómez et al., 2013; Pereira-Manrique and Reyes, 2013; Gil et al., 2014; Pedraza et al., 2016; Aguilar-Navarro et al., 2017; Delgado et al., 2017), Addenbrooke's cognitive examination (ACE) (Sarasola et al., 2005; Custodio et al., 2012; Herrera-Pérez et al., 2013), ACE-III (Bruno et al., 2017), and Addenbrooke's cognitive examination-revised (ACE-R) (Torralva et al., 2011; Muñoz-Neira et al., 2012a; Ospina, 2015), as well as complimentary tools designed to measure specific domains such as the memory alteration test (M@T) (Custodio et al., 2014, 2017a), INECO frontal screening (IFS) (Torralva et al., 2009; Ihnen Jory et al., 2013; Custodio et al., 2016), and frontal assessment battery (FAB; Dubois et al., 2000).

For use in LA, a BCS tool should be adapted, standardized, and validated for the region (Dua et al., 2011). To reflect the sociodemographic characteristics of older adults in LA, which include high indices of illiteracy and the presence of indigenous populations, data should also be available for individuals from both urban and rural areas and with various levels of education, including illiterate individuals (Carnero-Pardo, 2014; UNESCO, 2015, 2017; Instituto Nacional de Estadística e Informática., 2018). Although several reviews are available for BCSs in other countries and continents (Paddick et al., 2017; De Roeck et al., 2019; Magklara et al., 2019), no critical BSC-related reviews have been published for LA populations. Therefore, the objective of this article is to review studies on BCS designed to discriminate between normal cognition and mild cognitive impairment (MCI) or dementia in Spanish-speaking individuals over the age of 50. We also discuss the diagnostic accuracy of BCS methods designed to discriminate between amnestic MCI (aMCI) or early AD and normal cognition in Spanish-speaking individuals over the age of 50 years.

## Methods

### Search Criteria

Suitable published studies were identified by searching the following databases: Medline, Biomed Central, Embase, Scopus, Scirus, PsycINFO, LILACS, and SciELO. Three of the authors (RM, MM, and LD) independently searched for articles associated with the following terms in English: [(Dementia) OR (Cognitive Impairment)] AND [(Screening) OR (questionnaires) OR (brief cognitive screening) OR (validity)] AND [(“Latin America” OR “Hispanic American” OR “South America” OR “Caribbean” OR “Latinos” OR “Mexico” OR “Colombia” OR “Argentina” OR “Chile” OR “Peru”)]. Next, the authors performed a search for the same terms in Spanish. The authors searched for articles published between 1953 and July 30, 2018, considering that PRISMA for Systematic Review (Moher et al., 2015).

### Inclusion/Eligibility Criteria

Study titles and abstracts were independently read by RM, MM, and LD to exclude duplications or articles unrelated to the validation of a BCS. After obtaining the full text of each article, the following inclusion criteria were applied: the study was carried out on Spanish-speaking individuals over the age of 50; a standardized protocol was used for the diagnostic process; the study was carried out in a memory clinic or research center based in LA; BCSs took less than 15 min to administer; psychometric measures (content validity, criterion validity, internal consistency, and diagnostic validity) were available; and the BCS was compared with gold-standard diagnostic criteria (including the last three versions of the Diagnostic and Statistical Manual of Mental Disorders [DSM]; diagnosis by a neurologist, psychiatrist, or geriatrician; or diagnosis based on a detailed neuropsychological evaluation). BCSs were included irrespective of the type of dementia evaluated.

### Exclusion Criteria

Studies were excluded if they were based on tests administered by telephone, self-evaluations, and caregiver or informant interviews, or if the analysis was limited to a simple correlation between the BCS and a previously established neuropsychological battery. Studies were excluded if the tests evaluated were detailed neuropsychological batteries or screenings for cognitive decline secondary to depression, traumatic brain injury, or cerebrovascular disorder.

### Selection of Studies

The full text of every article was read by NC and CA. These authors analyzed the data on the process of translating or culturally adapting the tool when the original BCS was written in a language other than Spanish. The quality of the translation and cultural adaptation procedures reported were evaluated using the Manchester translation reporting questionnaire (MTRQ) and Manchester cultural adaptation reporting questionnaire (MCAR), respectively. Both the MTRQ and MCAR (Landis and Koch, 1977) are seven-point scales developed by the Center for Primary Care Research at the University of Manchester to quantify the quality of procedures reported for translation and cultural adaptation of neuropsychological evaluations. For BCSs originally written in Spanish, only the MCAR was applied. Studies were included in this analysis if they received a score of at least 2a on the MTRQ and MCAR. Given that there are several Spanish-language versions available for the MoCA, and that the original authors have not authorized these versions, we analyzed studies that met the above inclusion/exclusion criteria. When necessary, authors were contacted to obtain the full text of the study or additional unpublished data.

### Analysis and Evaluation of BCSs

NC, MM, and LD recorded specific data for each study, including the author name(s), year, and complete title of the publication; site where the research was performed (specifically, the location from which the study sample was recruited); study objectives; type of BCS used; cognitive domains evaluated, categorizing each study as a domain-specific or global evaluation; geographic location; institution that performed the research; type of dementia evaluated; and diagnostic utility. The quality of the BCS was evaluated according to the following properties: content validity, internal consistency, criterion validity, construct validity, reproducibility, diagnostic accuracy, floor/ceiling effects, and interpretability (see Table 1). Each evaluated property was scored separately by two authors (RM and MM) as positive, negative, or indeterminate. In general, a positive score was assigned if the characteristics of the property were mentioned in the study design and described in detail in the methods and analysis of results. A negative score was assigned if the characteristics of the property did not meet these criteria. A score of “indeterminate” was assigned if the characteristics of each property were mentioned in a general manner in the study design, but a detailed description was not included in the methods and analysis of results. A score of “indeterminate” was also assigned if the characteristics of the property were not mentioned or described in the methods or analysis of results.

**Table 1:** Evaluation criteria for studies on BCS.

**Type of criteria**	**Definition**	**Criteria for a positive score**
Content validity	Refers to the extent to which the BCS represents all aspects of the domains assessed (Streiner and Norman, 2003; Terwee et al., 2007). Includes description of the population to which the BCS is to be applied and evaluation of the BCS by dementia experts with at least two years of experience in cognitive and neuropsychological evaluation who responded to the content validity questionnaire. This questionnaire should contain the conceptual and operational definitions of the cognitive domains assessed and their respective indicators. The definition of each indicator should indicate how to administer the measure and provide instructions for scoring the corresponding domain. The experts should also be asked to assess the capacity of each subtest to evaluate the given cognitive domain, the capacity of each subtest to measure the corresponding indicator, and the clarity of the administration and scoring instructions. The experts should have the opportunity to provide observations and commentary as a basis for consensus discussions within the research team (which should consist of neurologists, geriatricians, psychiatrists, neuropsychologists, and/or other specialists) as well as discussions regarding suggestions and changes to the initially-proposed version of the tool.	A positive score was assigned if the authors: -Described the population and used a content validity questionnaire to obtain approval by expert consensus.
Internal consistency	Refers to the homogeneity of items within a cognitive domain on the BCS, the correlation between domain and composite scores, and the assessment of whether these measures truly evaluate the same concept (Streiner and Norman, 2003; Terwee et al., 2007). Internal consistency should be measured using Cronbach's alpha. The effect of successively removing single items from the BCS on the Cronbach's alpha value should be evaluated.	A positive score was assigned if Cronbach's alpha was ≥070.
Criterion validity	Refers to the extent to which a BCS score is related to another applicable measure, ideally a “gold standard.”	A positive score was assigned if there was a precise sample selection method, a detailed description of the sample, and positive correlation between the BCS and a “gold standard.”
Construct validity	Refers to the use of indirect evidence to measure validity in the event that a “gold standard” is not available (Terwee et al., 2007).	A positive score was assigned if the total score on the BCS and its cognitive domains were correlated with the MMSE, functional scales, or clinical dementia rating (CDR) scores in the individuals evaluated and if Spearman's correlation coefficient was applied when the data distribution was not normal.
Reproducibility	Refers to the degree to which repeated measures in stable patients produce the same results (Terwee et al., 2007). The concept of reproducibility includes two elements: agreement and reliability. Agreement reflects the extent to which repeated measures produce the same results, which may be expressed as the standard error of measurement (SEM) or a Bland-Altman plot (de Vet et al., 2006). Reliability assures us that patient groups evaluated with the BCS can be distinguished from controls or other patient groups despite measurement error. Reliability can be evaluated using a statistic such as the intraclass correlation coefficient (ICC) (McGraw and Wong, 1996).	A positive score was assigned if the ICC ≥0.70.
Diagnostic accuracy	Diagnostic accuracy was evaluated according to the results of the receiver operating characteristic (ROC) curve analysis. ROC analysis can be used to identify cut-off points and calculate the area under the curve (AUC) in order to assess the sensitivity, specificity, and predictive value of the various cut-off points (Streiner and Norman, 2003; Bravo-Grau and Cruz, 2015).	A positive score was assigned if the if AUC was ≥0.70.
Floor and ceiling effects	A floor or ceiling effect was determined to be present if more than 15% of patients obtained the lowest (floor) or highest (ceiling) scores possible. When these effects are present, patients above or below these limits cannot be distinguished from one another, and change or variability cannot be measured (Terwee et al., 2007).	A positive score was assigned if these effects were absent.
Interpretability	Refers to the capacity to assign qualitative meaning to the quantitative scores, so that the BCS results can be interpreted. Adequate information should be available to determine whether a score or a change in score is clinically significant (Terwee et al., 2007).	A positive score was assigned if the authors provided statistics for: -A reference population (controls) -Subgroups of relevant patients (dementia, subtype of dementia, MCI).

## Results

### Studies Included

**Figure 1:**
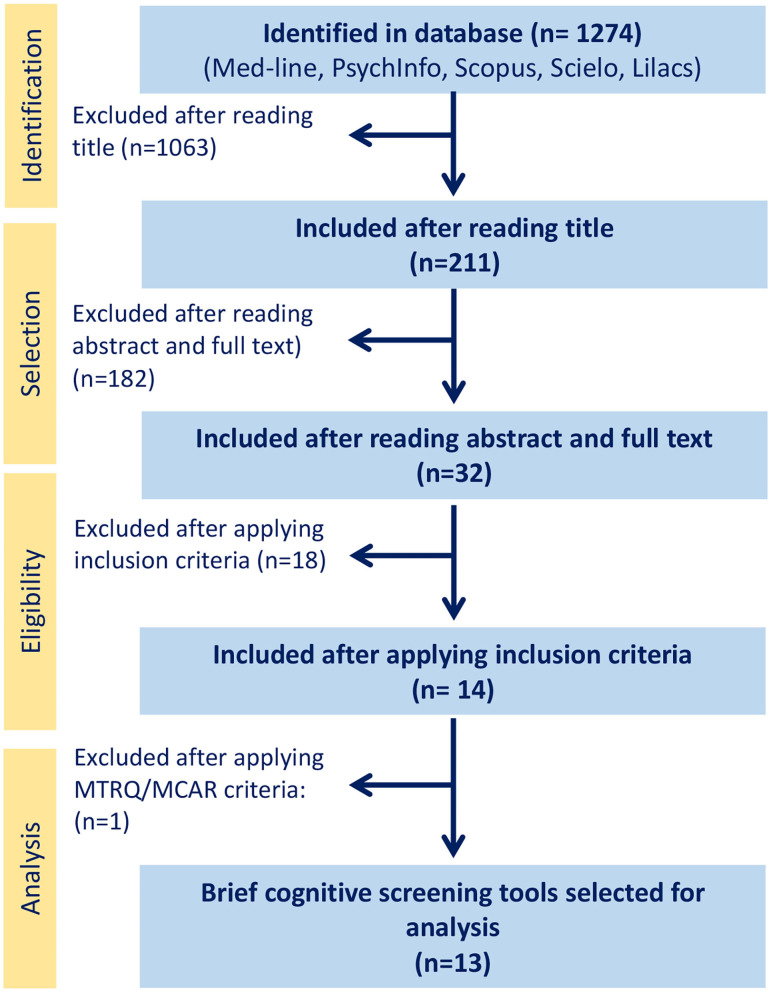
Flowchart of the study selection process.

After reading the abstract and full text of each study, our search identified 32 studies on BCSs to detect cognitive decline or dementia carried out in LA. The selection process is depicted in the flowchart in Figure 1. After applying the inclusion criteria, 18 studies were excluded for the following reasons: two for the absence of a standardized diagnostic protocol for study sample selection (Iturra-mena, 2007; Cantor-Nieto and Avendaño-Prieto, 2016); two for the use of another BCS, such as the Leganés cognitive test (Gómez et al., 2013) or the MEC Lobo, as the diagnostic criterion for study sample selection (Serrani Azcurra, 2013); six for the use of a tool that takes more than 15 min to administer, such as ACE (Sarasola et al., 2005; Custodio et al., 2012; Herrera-Pérez et al., 2013) or ACE-R (Torralva et al., 2011; Muñoz-Neira et al., 2012a; Ospina, 2015); three for not reporting diagnostic accuracy measures (Rosselli et al., 2000; Labos et al., 2008; Pedraza et al., 2014); four for reporting only one diagnostic accuracy measure (Ostrosky-Solís et al., 2000; Quiroga et al., 2004; Franco-Marina et al., 2010; Oscanoa et al., 2016); and one for including patients with depression in the sample (Fiorentino et al., 2013). After applying the MTRQ/MCAR to the 14 remaining publications, a study validating the Manos version of the clock-drawing test was also excluded (Custodio et al., 2011) as the translation and cultural adaptation process was not described. Finally, 13 publications were selected for the definitive analysis (see the description in Table 2), five of which evaluated the MoCA (Pereira-Manrique and Reyes, 2013; Pedraza et al., 2016; Aguilar-Navarro et al., 2017; Delgado et al., 2017), three the IFS (Torralva et al., 2009; Ihnen Jory et al., 2013; Custodio et al., 2016), two the M@T (Custodio et al., 2014, 2017a), and one the memory, fluency, and orientation (MEFO) test (Delgado Derio et al., 2013); the Phototest (Russo et al., 2014) and the last one, about Memory Binding Test (MBT) (Roman et al., 2016). Of these publications, three were from Chile (Delgado Derio et al., 2013; Ihnen Jory et al., 2013; Delgado et al., 2017), three from Peru (Custodio et al., 2014, 2016, 2017a), three from Colombia (Pereira-Manrique and Reyes, 2013; Gil et al., 2014; Pedraza et al., 2016), three from Argentina (Torralva et al., 2009), and one from Mexico (Aguilar-Navarro et al., 2017). The tests assessed global cognitive function, memory, and executive function. All the publications assessing the MoCA (Pereira-Manrique and Reyes, 2013; Gil et al., 2014; Pedraza et al., 2016; Aguilar-Navarro et al., 2017; Delgado et al., 2017) and the MEFO test (Delgado Derio et al., 2013) compared the performance of control groups, patients with MCI, and patients with dementia. Two (Torralva et al., 2009; Custodio et al., 2016) of the three studies assessing the IFS test also evaluated the capacity of this test to detect FTD and early-stage ADD. The studies assessing the M@T (Custodio et al., 2014, 2017a) and the Phototest (Russo et al., 2014) evaluated the capacity of the test to discriminate patients with aMCI and early ADD from controls; meanwhile the MBT (Roman et al., 2016) comparing control and patients with MCI. All studies were clinically defined but no biomarkers were used to define whether these patients had ADD.

**Table 2:** Description of BCS for early dementia detection in Spanish-speaking LA older adults.

**BCS**	**Year**	**Author**	**Country**	**Site**	**Cognitive function measured**	**Study objective**	**Type of cognitive impairment studied**
IFS^a^	2009	Torralva T, et al.	Argentina	Memory clinic	Executive function	Demonstrate that the IFS can discriminate early executive function deterioration in patients with BvFTD^i^ from patients with early-stage ADD^j^	BvFTD and ADD
MEFO^b^	2012	Delgado Derio C, et al.	Chile	Memory clinic	Global cognitive efficiency	Validate the Spanish-language MEFO as a dementia and MCI^k^ screening test and compare results with MMSE^l^	MCI and dementia
MoCA^c^	2013	Pereira-Manrrique F, et al.	Colombia	Primary Health Center	Global cognitive efficiency	Establish reliability and validity measures for the MoCA as a dementia and MCI screening tool in adults over the age of 65 years living in Bogota	MCI and dementia
IFS-Ch^d^	2013	Ihnen Jhory J, et al.	Chile	Memory clinic	Executive function	Adapt the IFS to the Chilean cultural context and evaluate the psychometric properties and diagnostic accuracy of the tool in patients with dementia and controls	Dementia
MoCA-S^e^	2014	Gil L, et al.	Colombia	Memory clinic	Global cognitive efficiency	Validate the MoCA-S in Colombia and propose cut-off points	MCI and dementia
M@T^f^	2014	Custodio N, et al.	Peru	Memory clinic	Episodic and semantic memory	Evaluate the validity of the M@T to discriminate between patients with normal cognition and patients with amnestic MCI or early-stage ADD	Amnestic MCI and ADD
Phototest	2014	Russo MJ, et al.	Argentina	Memory clinic	Memory and verbal fluency	Estimate the diagnostic accuracy of the Phototest for cognitive impairment and dementia and to compare with other screening tests in an Argentine population	Amnestic MCI and ADD
MoCA	2016	Pedraza L, et al.	Colombia	Community	Global cognitive efficiency	Evaluate the reliability and criterion and discriminant validity of the MoCA in adults over the age of 50 years with various levels of education living in Bogota	MCI and dementia
IFS	2016	Custodio N, et al.	Peru	Memory clinic	Executive function	Evaluate the clinical utility of the IFS to differentiate: a) patients with dementia from controls and b) patients with BvFTD from patients with ADD, and compare the results to the performance of the FAB^m^	BvFTD and ADD
MBT^g^	2016	Roman F, et al.	Argentina	Memory clinic	Memory	Determine the reliability and validity of the Spanish version of the MBT in South America as a tool for early detection of MCI in Argentina.	MCI
M@T	2017	Custodio N, at al.	Peru	Adult day care	Episodic and semantic memory	Evaluate the validity of the M@T to discriminate among controls, patients with amnestic MCI, and patients with early-stage ADD in a sample of individuals with a low education level	Amnestic MCI and ADD
MoCA-S	2017	Aguilar-Navarro S, et al.	Mexico	Memory clinic	Global cognitive efficiency	Validate the MoCA-S in Mexico as a tool to screen for MCI and dementia in Mexican older adults	MCI and dementia
MoCA- E^h^	2017	Delgado C, et al.	Chile	Adult day care	Global cognitive efficiency	1) Study the psychometric properties of the MoCA-E; 2) study the discriminative validity of the MoCA-E for diagnosis of MCI and mild dementia in a Chilean population and compare the results to the performance of the MMSE; 3) Identify the items on the MoCA that are most sensitive in detecting MCI and dementia	Amnestic MCI, non-amnestic MCI, and dementia

a *IFS, INECO Frontal Screening*.

b *MEFO, Memory, Fluency and Orientation*.

c *MoCA, Montreal Cognitive Assessment*.

d *IFS-Ch, INECO Frontal Screening—Chile*.

e *MoCA-S, Montreal Cognitive Assessment in Spanish*.

f *M@T, Memory Alteration Test*.

g *MBT, Memory Binding Test*.

h *MoCA-E, MoCA en español*.

i *BvFTD, behavioral variant frontotemporal dementia*.

i *ADD, Alzheimer's disease dementia*.

k *MCI, Mild cognitive impairment*.

l *MMSE, Mini-Mental State Examination*.

m *FAB, Frontal Assessment Battery*.

### Analysis and Quality of the BCS Tools

A summary of the psychometric properties of the BCS tests for early dementia detection in older Spanish-speaking adults in LA is shown in Table 3.

**Table 3:** Psychometric properties of BCS for early dementia detection in Spanish-speaking LA older adults.

**BCS**	**Content**	**Internal**	**Criterion**	**Construct**	**Agreement**	**Reliability**	**Diagnostic**	**Floor/ceiling**	**Interpretability**
	**validity**	**consistency**	**validity**	**validity**			**accuracy**	**effect**	
IFS^a^, Torralva	+^*^	+	+	+	ND	+	+	ND	+
MEFO^b^, Delgado Derio	-^**^	–	+	+	ND	ND	+	ND	+
MoCA^c^, Pereira	?^***^	ND	?	+	ND	ND	+	ND	+
IFS-Ch^d^, Ihnen Jory	+	+	+	+	ND	ND	+	ND	+
MoCA-S^e^, Gil	–	+	+	+	ND	ND	+	ND	+
M@T^f^ 2014, Custodio	–	ND	+	ND	ND	ND	+	ND	+
Phototest, Russo	ND	ND	ND	+	ND	ND	+	ND	+
MoCA, Pedraza	–	+	+	–	+	+	+	ND	+
IFS, Custodio	–	ND	+	ND	ND	ND	+	ND	+
MBT^g^, Roman	ND	ND	+	+	ND	ND	+	ND	+
M@T 2017, Custodio	?	+	+	+	ND	ND	+	ND	+
MoCA-E^h^, Aguilar-Navarro	–	+	+	+	ND	ND	+	ND	+
MoCA-S, Delgado	–	+	+	+	ND	ND	+	ND	+

* +, *positive*;

** *negative*,

*ND, no data available*;

*** *?, indeterminate*.

a *IFS, INECO Frontal Screening*.

b *MEFO, Memory, Fluency and Orientation*.

c *MoCA, Montreal Cognitive Assessment*.

d *IFS-Ch, INECO Frontal Screening—Chile*.

e *MoCA-S, Montreal Cognitive Assessment in Spanish*.

f *M@T, Memory Alteration Test*.

g *Memory Binding Test*.

h *MoCA-E, MoCA en español*.

#### Content Validity

Only two of the publications (Torralva et al., 2009; Ihnen Jory et al., 2013) provided an adequate description of the study and the process of adaptation to the local cultural context before the administration of the test. The validity of the BCS was assessed by consulting with a group of experts using a content validity questionnaire.

#### Internal Consistency

A Cronbach's alpha of 0.69 was reported for the MEFO test (Delgado Derio et al., 2013). This statistic was not reported in one of the studies evaluating the MoCA in Colombia (Pereira-Manrique and Reyes, 2013) or in the studies assessing the IFS (Custodio et al., 2016) or M@T (Custodio et al., 2014) in Peru. The studies that evaluated the Phototest (Russo et al., 2014) and the MBT (Roman et al., 2016) also did not report on internal consistency.

#### Criterion Validity

Twelve of the 13 studies met the gold standard for this measure, the exception being the study by Pereira-Manrique and Reyes (2013), that was rated as “indeterminate” as the methods only mentioned in a general manner that the diagnosis of dementia was based on DSM-IV-TR criteria, and not data available in Phototest's study (Russo et al., 2014).

#### Construct Validity

Only two studies failed to provide data (Custodio et al., 2014, 2016) correlating the BCS with the MMSE or other global functioning scale such as the clinical dementia rating (CDR).

#### Reproducibility

Most of the studies failed to provide the data necessary to evaluate this characteristic. Only one study (Pereira-Manrique and Reyes, 2013) provided adequate data on concordance and reliability. The study by Torralva et al. (2009) only presented data on reliability.

#### Diagnostic Accuracy

All the studies met diagnostic accuracy criteria for various cut-off points.

#### Floor and Ceiling Effects

None of the 13 studies reported results for this characteristic.

#### Interpretability

All the investigators included a comparison group (controls vs. patients with dementia, controls vs. patients with MCI) to evaluate the results of BCSs, and the diagnostic accuracy measures allowed the authors to establish cut-off points to discriminate between the groups.

#### Measures of Diagnostic Accuracy

To analyze the diagnostic accuracy measures for the BCS to discriminate MCI and dementia from normal cognition in Spanish-speaking patients over the age of 50, we assessed performance according to age and education level, requesting additional information if the publication did not provide this data. Olga Pedraza (Pedraza et al., 2016) responded *via* email with the number of cases per group evaluated, as well as the age and education level of each group (control, MCI, and dementia) (see Table 4).

**Table 4:** Education level and age of study samples and diagnostic accuracy measures for BCS to detect MCI and dementia in Spanish-speaking adults over the age of 50 years.

**BCS**	**Average years of education of sample ± *SD***	**Average age ± *SD***	**Sample size**	**Cut-off point**	**Se**	**Sp**	**AUC**	**Comments**
IFS^a^, Torralva	Control: 14.5 ± 2.2 BvFTD^i^: 16.3 ± 3.1 ADD^j^: 14.5 ± 3.6	69.2 ± 8.9 70.5 ± 6.1 77.6 ± 5.2	C^l^/FTD/ADD 26/22/25	25/30 19/30	96.2% 72%	91.5% 81.3%	0.980 (C vs. D) 0.776 (FTD vs. ADD)	
MEFO^b^, Delgado Derio	Control: 12 ± 4 MCI^k^: 9 ± 5 Dementia: 12 ± 4	70 ± 7 75 ± 7 73 ± 7	C/MCI/D 118/47/49	7 9	86% 68%	96% 76%	0.971 (C vs. D) 0.776 (C vs. MCI)	
MoCA^c^, Pereira	Completed elementary school: 14% Some elementary school: 41% Illiterate: 38%	Not reported	C/MCI/D 59/47/49	21.5/30 19.5/30 14.5/30	88% 100% 58%	20% 59% 20%	0.92 (>11 years/education) 0.83 (5–10 years/education) 0.77 (<5 years/education)	Limitations in populations with low education level
IFS-Ch^d^, Ihnen Jory	Control: 11.9 ± 4.5 Dementia: 9.7 ± 4.7	70.9 ± 8.2 74.1 ± 9.2	C/MCI/D 30/0/31	18/30	90%	87%	0.951	
MoCA-S^e^, Gil	Control: 14 ± 4.7 MCI: 13 ± 5.1 Dementia: 11 ± 5.1	68 ± 10.38 65 ± 13.4 73 ± 7.5	C/MCI/D 84/26/83	23/30	89%	79.8%	0.93	Limitations in populations with low education level
M@T^f^ 2014, Custodio	Control: 6.99 ± 3.15 MCIa: 6.49 ± 2.73 ADD: 6.56 ± 2.87	69.97 ± 4.04 71.09 ± 4.20 74.16 ± 3.73	C/MCIa/ADD 180/45/90	27/50 37/50	100% 98.33	97.78% 98.89%	(MCIa vs. ADD) 0.9986 (C vs. MCI)	Utility of total score greater than that of individual domains
Phototest, Russo	Control: 9.97 ± 3.59 MCIa: 8.66 ± 4.15 ADD: 9.05 ± 3.99	74.17 ± 6.39 73.70 ± 6.66 75.41 ± 5.14	C/MCIa/ADD 30/61/56	≤ 27 ≤ 30	89.29% 85.25%	96.67% 90.00%	0.97 (C vs. ADD) 0.93 (C vs. MCI)	The analysis adjusted for age and education did not change the diagnostic accuracy of the Phototest.
MoCA, Pedraza	Control: 8.33 ± 5.84 MCI: 6.39 ± 5.17 Dementia: 3.56 ± 2.81	66.00 ± 7.87 69.91 ± 7.87 74.28 ± 8.18	C/MCI/D 153/168/105	22–23/30 17–18/30	83.3% 80.4%	30.6% 55.2%	*0.76 (C vs. MCI) *0.81 (MCI vs. D)	*Cut-offs for patients with less than elementary education
IFS, Custodio	Control: 11.75 ±2.4 BvFTD: 11.73 ± 2.66 ADD: 11.8 ± 2.8	70.15 ± 2.85 67.08 ± 4.04 73.57 ± 3.8	C/FTD/ADD 48/34/35	23.5/30 19.5/30	97.1% 94.1%	97.1% 94.2%	0.990 (C vs. D) 0.980 (FTD vs. ADD)	
MBT^g^, Roman	Control: 11.5 ± 4.1 MCI: 10.5 ± 1.2	67.5 ± 8.3 65.3 ± 5.4	C/MCI/D 46/42/0		69%	88%	0.89 (C vs MCI)	MBT standardization was performed by age and education in the normal healthy population
M@T 2017, Custodio	Control: 2.57 ± 1.45 MCIa: 2.53 ± 1.46 ADD: 2.65 ± 1.28	69.53 ± 4.11 71.09 ± 4.20 74.18 ± 3.81	C/MCIa/ADD 121/45/81	26/50 35/50	100% 99.1%	97.5% 91.1%	0.9960 (MCIa vs. ADD) 0.9956 (C vs. MCI)	Cut-offs should be adjusted for education level
MoCA-E^h^, Aguilar-Navarro	Control: 12.4 ± 3.7 MCI: 10.0 ± 5.6 Dementia: 9.5 ± 5.8	69.91 ± 1.11 75.15 ± 6.23 81.70 ± 5.88	C/MCI/D 59/52/57	26/30	80%	75%	0.886	Cut-offs do not need to be adjusted for age or education level
MoCA-S, Delgado	Control: 11.4 ± 4.2 MCIa: 9.2 ± 3.8 MCIna: 12.0 ± 4.3 Dementia:11.3 ± 4.7	72.3 ± 5.4 75.3 ± 7.8 74.3 ± 7.9 75.1 ± 8.2	C/MCIa^m^/ MCIna^n^/D^o^ 104/24/24/20	21/30 20/30	75% 90%	82% 86%	0.823 (C vs. MCIa), attention 0.916 (C vs. D), delayed memory	Large effect of education level; cut-offs for most stable domains shown

a *IFS, INECO Frontal Screening*.

b *MEFO, Memory, Fluency and Orientation*.

c *MoCA, Montreal Cognitive Assessment*.

d *IFS-Ch, INECO Frontal Screening – Chile*.

e *MoCA-S, Montreal Cognitive Assessment in Spanish*.

f *M@T, Memory Alteration Test*.

g *Memory Binding Test*.

h *MoCA-E, MoCA en español*.

i *BvFTD, behavioral variant frontotemporal dementia*.

j *ADD, Alzheimer's disease dementia*.

k *MCI, Mild cognitive impairment*.

l *C, Control*.

m *MCIa, Amnestic mild cognitive impairment*.

n *MCIna, Non-amnestic mild cognitive impairment*.

o *D, Dementia*.

In general, the studies analyzed involved study samples with an average of 10 years of education. Two studies involving the MoCA test (Pereira-Manrique and Reyes, 2013; Pedraza et al., 2016) and one involving the M@T (Custodio et al., 2014) reported a sample with an average of 5–10 years of education, and only one (Custodio et al., 2017a) reported a sample with an average of <5 years of education. One study, only compared controls and patients with dementia (Ihnen Jory et al., 2013) and other, only compared controls and patients with MCI (Roman et al., 2016). All of the studies involving the MoCA found that education level biased scores, with the exception of the study carried out in Mexico (Aguilar-Navarro et al., 2017). Finally, our review yields important differences in publishing data, mainly: population heterogeneity, heterogeneity in screening tests explaining by the difference in their application and validity, use of different diagnostic criteria limiting the combination of results of the publications to calculate cut-off points, sensibility, and specificity. Therefore, it was not possible to calculate metrics by gathering data of the published papers.

## Discussion

The objective of this study was to review evidence on the use of BCS test in detecting MCI and dementia in a LA population. The main conclusions of this review were that relatively studies have evaluated BCSs with adequate diagnostic accuracy measures in LA populations, and most of the BCS tests were validated in a memory clinic setting. Finally, the validated BCSs studied differed in terms of the cognitive domains evaluated. A large number of screening tests for ADD are available; however, most are only validated in a memory clinic setting and description of the psychometric properties of the instruments is limited. Analyzed tests, in particular, require further research. The MoCA is a promising BCS instrument, but shows low specificity in detecting early ADD.

After applying exclusion criteria, only 13 studies were retained for analysis in this review. These studies were performed in five countries. No studies on validated BCSs with adequate diagnostic accuracy measures performed in other countries in the region were identified. While there is a certain level of linguistic homogeneity in LA, studies on the standardization of neuropsychological batteries have suggested that significant differences exist among countries in terms of neuropsychological tests (Arango-Lasprilla, 2015). Given that reviews of BCS tools for other parts of the world have included up to 120 studies (De Roeck et al., 2019), our findings suggest that there is a notable lack of studies validating BCS tools in Spanish-speaking LA countries. Additional studies that include other LA countries are needed. Most of the studies identified were carried out in memory clinics, with only two being undertaken in adult day care centers (Custodio et al., 2017a; Delgado et al., 2017), one in a primary health center (Pereira-Manrique and Reyes, 2013) and one in the community (Pedraza et al., 2016). This finding is noteworthy given that results from memory clinics are not extrapolable to primary care centers and community samples. Indeed, BCSs have been reported to perform better in the community that in the clinical context (Paddick et al., 2017). However, some community-based studies have excluded individuals with significant sensory deficits, which may artificially inflate the diagnostic accuracy of the BCS (De Roeck et al., 2019). Additionally, studies in clinical contexts such as memory clinics or adult day care centers may include an elevated proportion of individuals with general frailty or medical conditions, which may also adversely affect the performance on the evaluated BCS test (Paddick et al., 2017; De Roeck et al., 2019). Owing to the high rate of underdiagnosis of dementia, there is a need for properly validated BCS tools in primary care centers. In general, results from community-based studies can reflect the true prevalence and severity of mild cognitive impairment and dementia, because when studies are conducted in specialized memory centers in clinics or general hospitals, the prevalence and severity tend to be higher. Furthermore, in this way, BCS tools could be used by primary care professionals, especially in rural populations.

All the studies included in this review analyzed the criterion validity and diagnostic accuracy of the BCS assessed, and most reported internal consistency and construct validity. Only one study addressed agreement (Pedraza et al., 2016), and two studies reported the reliability of the BCS (Torralva et al., 2009; Pedraza et al., 2016). None of the studies reported a floor/ceiling effect, which is consistent with the findings of previous reviews (Paddick et al., 2017; De Roeck et al., 2019; Magklara et al., 2019).

Most of the BCSs included in this review evaluated global cognitive efficiency, while a few assessed executive function (Torralva et al., 2009; Ihnen Jory et al., 2013; Custodio et al., 2016) or memory (Custodio et al., 2014, 2017a). Notably, vascular cognitive impairment, vascular dementia (VD), and mixed dementias are more common in LMICs than in higher-income countries; importantly, these dementias are best detected with global BCS tests or those that measure executive function (Dubois et al., 2000; Maestre et al., 2018). BCS tests that only evaluate memory have a better capacity to detect typical cases of Alzheimer's dementia, while BCS tests that evaluate executive function are superior for identifying disorders involving the prefrontal cortex, such as FTD and VD but are limited ability in their ability to identify other subtypes of dementia (Ihara et al., 2013).

MoCA is the most widely-used BCS in LA, especially in Colombia (Pereira-Manrique and Reyes, 2013; Gil et al., 2014; Pedraza et al., 2016). All the studies evaluating the MoCA carried out in Colombia reported that the education level biased the results, as did a study performed in Chile on a sample with a high level of education (Delgado et al., 2017). However, the Mexican authors (Aguilar-Navarro et al., 2017), who assessed a population with an education level similar to that of the Chilean sample, found no significant effect of age or education level, which differs from most results reported in the literature. A different study evaluated Brazilian patients with Parkinson's disease, 65% of whom had less than 8 years of education, as part of the LARGE-PD study. In that sample, there was a significant floor for some of the MoCA subtests (Tumas et al., 2016). For the attention subtest, which requires individuals to count backwards from 100, subtracting seven each time, most of the patients had failed by the fifth subtraction. Similar results were found for the repetition, verbal fluency, and abstraction subtests (Tumas et al., 2016). Moreover, in a sample of patients from the Andean region of Colombia, where the average education level was 4.8 years and where 8% of the people were illiterate, the MoCA subtests that were least biased by education were orientation, delayed recall, and repetition. In contrast, the part B subset of the Trail Making test was correctly performed by only 37% of people with an elementary-level education, and <30% of individuals with an elementary-level education and 7% of illiterate individuals correctly completed cube drawing (Gómez et al., 2013). Similarly, studies using MoCA in populations of Brazilian (Apolinario et al., 2018), Turkish (Yancar and Öscan, 2015), and Chinese (Zhang et al., 2019) origin have reported the influence of education level on MoCA cut-off points. A systematic review on the cultural validity of MoCA (O'Driscoll and Shaikh, 2017) and a critical review on BCS for older adults with low levels of education (Tavares-Júnior et al., 2019) suggest different cut-off points of MoCA according to education level. In this sense, taking into account the high proportion of people with low education and illiteracy in Latin America (UNESCO, 2015, 2017), certain MoCA tasks (drawing of the cube, denomination of dromedary and rhinoceros, and subtract backwards by seven from 100) could not be completed easily, increasing the real suspicion of cases with cognitive impairment. Also, it has been noted that MoCA cognitive domains reflect an educational gradient, including some form of language that might be primarily developed through schooling (Yancar and Öscan, 2015). Different mechanisms have been suggested to explain the relationship between education and cognition. It has been suggested that education may be a marker of other factors associated with cognition, including lower brain reserve may be related to low education.

A major strength of our study was that we used LA databases, including LILACS and SciELO. As some regional authors do not have access to high-impact journals, systematic reviews and meta-analyses often fail to include studies by these authors (Paddick et al., 2017; De Roeck et al., 2019; Magklara et al., 2019). Another strength of this study was the inclusion criteria and detailed evaluation of the diagnostic accuracy measures, as these elements are often overlooked in other publications (De Roeck et al., 2019; Magklara et al., 2019).

The limitations of this review include the scarcity of studies with sufficient information to discriminate between different types of dementia. The only studies to address different types of dementia were those carried out on IFS in Argentina (Torralva et al., 2009) and Peru (Custodio et al., 2016), which evaluated the capacity of this BCS to distinguish between ADD and behavioral variant FTD (BvFTD), and those on the M@T (Custodio et al., 2014, 2017a), which evaluated the ability of the tool to distinguish between aMCI and early ADD. This limitation is likely related to our inclusion criterion that the BCS administration time be no longer than 15 min, as evaluating different cognitive domains requires additional administration time to establish differential profiles of dementia. Therefore, the capacity of a BCS to discriminate among different types of dementia is questionable (De Roeck et al., 2019). On the other hand, some of the BCS tools only evaluated specific domains, such as episodic memory in the case of the M@T. When used for screening in the clinical context, it is possible that such BCS tests would fail to detect cases in which the initial sign of dementia do not include memory loss, such as, VD or FTD. Moreover, BCS tests that evaluate executive function, such as the IFS, may be ideal for detecting BvFTD and VD (Custodio et al., 2017b) but potentially at the cost of a decreased ability to detect AD. It is likely that cognitive screening tools that require a longer administration time, such as the ACE-R (Torralva et al., 2011; Muñoz-Neira et al., 2012a; Ospina, 2015), might be capable of discriminating among different types of dementia. However, our objective in this review was to evaluate BCS that can be used in a primary care setting.

A second limitation of this review was that we excluded BCSs based on caregiver or informant interviews, as tools such as the general practitioner assessment of cognition (GPCOG), informant questionnaire on cognitive decline in the elderly (IQCODE), Alzheimer disease 8 (AD8), or Pfeffer functional activities questionnaire (PFAQ) may be useful in the clinical setting. This resulted in the exclusion of tools to evaluate functional capacity, which may improve the ability of a test to detect cognitive decline (De Roeck et al., 2019; Magklara et al., 2019). Indeed, in the dementia field, most functional capacity evaluation tools are informant-based questionnaires owing to the frequent coexistence of cognitive decline with anosognosia (Muñoz-Neira et al., 2012b). This point is important, as evaluating instrumental activities of daily living is crucial for differentiating between MCI and dementia (De Roeck et al., 2019).

A third limitation is the inclusion of BCS tools only study in subjects over 50 years, even though there are dementias with an age-at-onset less than 50 years old like the Dominantly Inherited Alzheimer's Disease (DIAD) (McDade et al., 2018). Current evidence suggests that the same BCS are valid tool to diagnosis dementia regardless of aging of onset (Rossor et al., 2010). Nevertheless, it is important to highlight that most of BCS present low sensitivity to detect neurodegenerative disease at a preclinical stage. A final limitation relates to the fact that we have only included publications in Spanish, and probably we have not been able to access native aboriginal populations. In addition, there is the possibility of researchers publishing their findings in non-indexed journals.

In summary, this review showed that the M@T is the only BCS that has been evaluated in a group with a low education level; in LA the MoCA requires cultural adaptations and different cut-off points based on the level of education; the diagnostic validity of the IFS should be evaluated in populations with a low education level; finally, no publication to date has included an illiterate population.

## Conclusion

Our review on BCS tests for LA Spanish speaking population showed the need for additional studies in LA with adequate indices on the diagnostic validity of tools to screen for various stages of cognitive decline and different types of dementia. Moreover, the diagnostic accuracy of BCS tools need to be study in different settings (i.e., community, primary care and memory unit). Finally, low-level of education is beside age, one the main risk of dementia (Livingston et al., 2017). Unavailability of BCS properly validate in low -education and illiterate subjects is a strong barrier to diagnosis dementia in this population and there is an urgent need to validate BCS suitable for this population (Ortega et al., 2019).

## Author Contributions

NC and AS conceived the study. NC, RM, CA-D, and AS designed the study. RM, CA-D, MM, and LD collected the data. RM, MM, and CA-D organized the database. NC, RM, and AS wrote the first draft of the manuscript. NC, RM, and CA-D wrote sections of the manuscript. All authors contributed to manuscript revision, read, and approved the final version.

## Conflict of Interest

The authors declare that the research was conducted in the absence of any commercial or financial relationships that could be construed as a potential conflict of interest.
